# A pragmatic randomised controlled trial of preferred intensity exercise in depressed adult women in the United Kingdom: secondary analysis of individual variability of depression

**DOI:** 10.1186/s12889-019-7238-7

**Published:** 2019-07-12

**Authors:** Ioannis D. Morres, Anton Hinton-Bayre, Efthymios Motakis, Tim Carter, Patrick Callaghan

**Affiliations:** 1 Department of Physical Education and Sport Science, Exercise Psychology and Quality of Life Laboratory, University of Thessaly, Trikala, Greece; 2 School of Surgery, Ear Science Centre, University of Western Australia, Perth, Australia; 3 Yong Loo Lin School of Medicine, Cardiovascular Research Institute, National University of Singapore, Singapore, Singapore; 4 School of Health Sciences, University of Nottingham, Nottingham, UK; 5 School of Applied Sciences, London South Bank University, 103 Borough Road, London, SE1 0AA UK

**Keywords:** Exercise, Preferred intensity, Depression, Individual clinical significance

## Abstract

**Background:**

This study is a secondary analysis of the trial by Callaghan et al. (2011), which reported higher antidepressant effects for preferred intensity (*n* = 19) vs. prescribed intensity (*n* = 19) exercise of three sessions/week over four weeks in depressed women. In particular, the present study sought to examine whether greater clinically significant individual change/recovery was observed in the preferred compared to the prescribed exercise group.

**Methods:**

The reliable change index and the C_cutoff_ score criteria described by Jacobson and Truax (1991) were employed to determine clinical significance. These criteria examined if individual change in depression scores from pre- to post-intervention in the preferred intensity group were statistically significant beyond the standard error of difference derived from the active comparator prescribed group, and subsequently within a normal population range. Patients fulfilling the first or both criteria were classified as improved or recovered, respectively.

**Results:**

Post-intervention depression scores of six patients in the preferred intensity exercise group (32%) demonstrated statistically reliable improvement (*p* < 0.05) and recovery. Half of this subgroup started as moderately depressed. No patient demonstrated a reliable deterioration in depression. Due to a small sample size, it was impossible to determine whether patients on psychiatric medication or medication-free patients were equally benefited from preferred intensity exercise. Thirteen patients in the preferred intensity group (68%) displayed non-statistically significant change in post-intervention depression scores (*p* > 0.05), although eight of them showed a non-significant improvement in post-intervention depression scores and three could not technically show an improvement in depression due to floor effects (baseline depression within normal range).

**Conclusions:**

Preferred intensity exercise of three sessions/week over four weeks led almost a third of the patients to record scores consistent with recovery from depression. Health professionals may consider that short-term preferred intensity exercise provides clinically significant antidepressant effects comparing favourably to exercise on prescription.

## Background

Depression is a serious and disabling mental health disorder affecting women almost twice as often as men [1]. Worldwide projections for the year 2030 suggest that unipolar major depression will become the leading cause of disease burden [2]. Almost 20% of people in the UK aged 16 and over experience symptoms of depression and anxiety [3]. The largest increase in such symptoms is seen among women aged 45–64 [4].

In the UK, exercise on referral is an embedded treatment modality for mild or moderate depression [5] attributable to clinical evidence indicating the association of exercise with depression relief [6–9]. Yet, a number of depressed patients do not experience the beneficial effects of exercise in real-life (pragmatic) settings as they often show poor compliance to exercise on referral schemes due to the challenging clinical profile of depression (e.g., lack of motivation, time or resources) [10–12]. However, depressed patients who are referred into an exercise on referral scheme seem to be motivated in the initial referral stage from the referral endorsement through to the first appointment of the exercise referral scheme [10, 13]. Hence, it is possible that prescribed intensity itself could be related to poor compliance to exercise on referral, as it may not represent a well-tailored intervention for all depressed patients due to individual needs and preferences. Poor compliance involves consideration that various exercise programs including prescribed intensity exercise are not tailored to individual needs and preferences [14].

Prescribed intensity exercise represents a nomothetic model that refers to training at fixed intensities [15], and as such is untailored to the individual’s needs or preferences. Interestingly, early evidence demonstrates that people with mental health problems, including depression, when exercising at fixed intensities showed disturbed perceived exertion. Disturbed physical exertion is a state that is linked with negative cognitive, behavioural and affective responses [16–18]. Conversely, the ideographic model of preferred intensity exercise excludes fixed intensities, promoting training at preferred levels of perceived exertion and allowing “space” for individual variability [15]. The importance of individual variability with respect to the patient’s needs and preferences is highlighted by the guidelines of the National Institute for Health and Care Excellence (NICE) for the treatment of depression via physical activity ([5]; pp.8) or for the promotion of physical activity in primary care ([19]; pp.10). Similarly, guidelines of the Canadian Network of Mood and Anxiety Disorders (CANMAT) for exercise and depression have reported the importance of self-directed over practitioner-directed therapies because the involvement of patient preferences may bring about better treatment response [20].

Various studies suggest better affective response to preferred over prescribed intensity exercise in adult and adolescent general populations [21–27]. Also, various studies have reported the promising antidepressant role of preferred exercise in adult and adolescent depression [28–30]. Our research group has previously directly compared the preferred intensity vs. the prescribed intensity model in a pragmatic randomised controlled trial (RCT) with a community sample of depressed women. Using group-based statistics, we found a large statistically significant standardized mean difference (SMD) in post-intervention depression for the preferred intensity group over the prescribed intensity comparator group (SMD = − 0.71, CI 95% -1.50 to − 0.17) [31]. Pragmatic trials are essential for translational research, as they inform on the effectiveness of intervention in “real-life” by replicating routine practice conditions. This replication ensures high external validity by recruiting participants in usual care with minimum exclusion criteria, and by delivering the intervention in every day treatment settings with commonly available resources/staff [32–34].

However, RCTs with group statistics such as the study of Callaghan et al. [31] offer little guidance to the clinicians wishing to quantify individual treatment outcome. To this extent, clinicians are not aware of the patient or proportion of patients with an improvement or deterioration in the outcome of interest. Also, studies with group-based statistics do not take into account the reliability of the outcome measure, and the amount of change that might thus be attributable to measurement error.

In response to this, clinical significance analysis described by Jacobson and Truax (JT) [35] determines whether an individual’s retest score is significant beyond the standard error of measurement, and also, whether the score moves from ‘abnormal’ to within the ‘normal’ range. Individual-based analysis techniques serve to complement more traditional mean-based statistical/practical significance methods offering meaningful information to the clinician who is typically challenged by the diverse therapeutic needs of the patients.

The pragmatic RCT by Doose et al. [36] explored the clinical and individual antidepressant effects of an eight-week ideographic preferred intensity exercise model (*n* = 30) vs. the wait-for-intervention condition (*n* = 16) in patients diagnosed with a depressive episode. Pre- and post-intervention depression assessed via a self-report outcome [Beck Depression Inventory-II [BDI-II]; 37] showed 36.67, 16.67 and 46.67% of the preferred intensity group with scores consistent with depression recovery, improvement and non-significant change, respectively. When Doose et al. [36] rated pre- and post-intervention depression via a clinician-report outcome (Hamilton Rating Scale for Depression-17 [HAMD-17]; [37]), the preferred intensity patients with recovery coefficients increased to 63.33%, and with improvement and non-significant change dropping to 13.33 and 23.33%, respectively.

The important study of Doose et al. [36] did not clarify three aspects of the impact of preferred intensity exercise on depression, which are in the scope of the current study. First, Doose et al. did not employ the benchmark prescribed-intensity exercise as a direct comparator. Thus, it has to be explored if preferred vs. prescribed intensity exercise shows clinically significant greater antidepressant effects at the individual level. Second, the mixed-gender sample provided no gender-specific evidence, especially for depressed women who represent the largest group in depression treatment [38]. Third, participant recruitment was achieved through media advertisements setting into question the generalizability of the findings, as Blumenthal and Ong [39] report that volunteers for exercise trials for depression are typically motivated to exercise.

This study aimed to re-evaluate the antidepressant effects of a short-term preferred intensity exercise model in direct comparison to a prescribed intensity exercise model among depressed women recruited via health services as reported in the pragmatic RCT of Callaghan et al. [31], by assessing the clinical significance of changes in individual depression scores.

## Method

### Participants, interventions and outcomes

Callaghan et al. [31] recruited women aged 45–65, as this group shows increasingly high rates of mental health disorders including depression [4]. Women were included if they were living in the community, and being monitored by, or receiving treatment for depression in health services. Women were excluded if they were unable to participate due to any injury or physical health problem. Prior to commencing the programme, they were asked to check with their General Practitioner and provide a consent form. A total of 43 women signed a consent form representing 63% of the eligible sample. Similar response rate (65%) has been recorded by a recent systematic review for exercise for depression [9].

Participants were randomly assigned to an experimental (*n* = 22) or active comparator group (*n* = 21), and then exercised in groups of up to five according to a preferred or prescribed intensity training regime on a treadmill three times/week for four weeks in public gyms free of charge. The preferred intensity group selected the intensity at a personal-preference on the basis of preferred exertion. The prescribed intensity group exercised at fixed intensities as defined by national guidelines (see Callaghan et al. [31]). During training, Heart Rate (HR) was measured with HR monitors (POLAR-FT1) and exertion with the Borg’s 6–20 Rating Perceived Exertion scale (RPE) which asks respondents to point out how hard they exercise (from 6 = no exertion to 20 = maximal exertion) [16].

Depression was measured with the BDI-II by Beck et al. [40], which was completed by both groups pre- and post-intervention, with higher scores indicating elevated severity of depression. The BDI-II is a widely used self-report scale for depression with good psychometric properties. Based on outpatients (*n* = 500) recruited via four clinics, Beck et al. reported using receiver operating characteristic analysis the following cut scores of severity: 0–13 minimal or no depression; 14–19 mild depression; 20–28 moderate depression; and 29–63 severe depression [40]. Both the preferred and prescribed groups were supervised by the same exercise therapist, and received manualised psychosocial support for exercise by the same health psychologist showing no post-intervention differences in the relevant social support outcome. Also, 2 and 3 patients dropped out in the preferred (10%) and prescribed group (14%), respectively. A £10 thank you voucher was given to all participants at discharge. Additional details can be found in Callaghan et al. [31].

### Secondary data analysis

We performed secondary analysis of the trial of Callaghan et al. [31] to explore if greater clinically significant individual change in post-intervention depression (BDI-II) was observed in the preferred vs. the prescribed exercise group. We used the JT analysis [35]. The JT includes the Reliable Change Index (RCI) methodology that seeks to determine if an individual’s change score is statistically significant. As the simplest and most popular RCI method, it determines whether a single change score sufficiently exceeds an error measure referred to as the standard error of difference or SED (RCI=Individual Change score/SED). The SED is a derivation of the standard error of the mean, but for difference scores. The SED describes the spread of the distribution of retest scores that would be expected given no change and can be derived from a comparator or normative retest group when the test and retest variances and reliability are known. The JT is a widely used analysis that shows comparable accuracy to other methods when practice effects are absent [41], as they were in the active comparator prescribed-exercise group.

Accordingly, we employed the BDI-II baseline and retest variances and test-retest reliability coefficient from our prescribed intensity comparator (Pearson *r* = .88; *p* < 0.05). In this vein, we aimed to search for individual depression differences of the preferred intensity group in excess of what would be expected under the comparator of prescribed intensity. RCI can be expressed as a standard (Z) score; if the Z score exceeds the value of ±1.96, a change is considered significant at the 95% level of confidence (two-tailed). Since each group in our study consisted of < 50 patients (*N* = 19), we treated the RCI as a t value instead, changing the significance level according to degrees of freedom, e.g. df = N-1 = 18. The level of significance in our study was set at *p* < 0.05 two-tailed, such that a negative (−) or positive (+) t(18) = 2.101 indicated a statistically significant improvement or deterioration of post-treatment BDI-II score, respectively.

Aside from assessing for a change in pre to post scores, the JT approach to clinical significance also examines whether a post-treatment score is likely to fall within the desired or normal population range. The JT approach sets the cut-off (C_cutoff_) midway between the dysfunctional and normal population distributions, provided that the latter data are available. In our analysis, normal population BDI-II data were retrieved from Seggar et al. [42]. A treatment response score below the C_cutoff_ is considered more likely to belong within the normal rather than the dysfunctional population. The C_cutoff_ is determined through subtraction of the normal population mean from the dysfunctional one (*M*_*0*_*–M*_*1*_), divided by the variance of the two population distributions. In case of differences between variances, the Standard Deviations of the dysfunctional and normal distributions (*σ*_*1*_ and *σ*_*0*_) are factored in as: C_cutoff =_ $\frac{\left(\mathrm{M}0\upsigma 1+\mathrm{M}1\upsigma 0\right)}{\upsigma 1+\upsigma 0}$. In our study, calculation of C_cutoff_ yielded a score of 14.39 (so less than 15) on the BDI-II.

By combining the C_cutoff_ and RCI t values, individual treatment response (post-intervention BDI-II scores) may be classified as follows in our study:

(1) Recovered: statistically significant decline in depression, now falling in the normal range; (2) Improved: statistically significant decline in depression, still outside the normal range;(3) Deteriorated: statistically significant increase in depression, still outside the normal range;(4) Unchanged: non-statistically significant change in depression, still in abnormal range.

Taking into account that pragmatic RCTs employ broad inclusion criteria with no symptom severity cut-points [33], a number of participants of our pragmatic RCT may record baseline depression scores within the normal range, and thus, an inability to reach recovery coefficients due to floor effects. Therefore, the latter classification in our study (4-Unchanged) may also refer to either: non-statistically significant change in depression, still in the normal range; or non-statistically significant change in depression, now falling from the normal to the abnormal range.

In the preferred intensity group, differences between recovered and non-recovered subgroups with respect to age were examined with the independent t-test and with respect to RPE and actual HR responses to exercise with the Wilcoxon rank-sum test. Spearman rank-order correlation coefficients explored associations between RPE and actual HR for each subgroup (recovered and non-recovered patients) in the preferred intensity group.

## Results

At baseline, the preferred intensity and the prescribed intensity groups showed moderate to severe depression (preferred intensity group: BDI-II mean = 26.5 ± 10.7; prescribed intensity group: BDI-II mean 30.5 ± 12.0). No significant differences between groups in baseline depression were found (see [31]). Three cases in each exercise group recorded BDI-II scores within the normal range (< 15 on the BDI-II scale).

Six patients in the preferred intensity group (32%), including two severely, three moderately and one mild depressed patient, recorded a significantly greater change in post-intervention BDI-II scores vs. the prescribed intensity comparator. These changes indicated statistically significantly reductions beyond the SED that was derived from the comparator group. The same six patients of the preferred intensity group also recorded recovery coefficients as their reduced post-intervention depression scores fell within the normal range (< 15 on the BDI-II scale).

Thirteen patients (68%) of the preferred intensity group showed no significant change in depression post-intervention, however, three had minimal depression and thus could not technically show a significant improvement due to floor effects (baseline depression in the normal range). Hence, the actual proportion with no significant improvement in depression is dropping from 68 to 53%, which is now equal to ten patients; noteworthy, eight of them showed non-significant improvement in depression. Finally, there was no statistically significant within group mean change in BDI-II scores in the prescribed exercise comparison group [31], supporting the appropriateness of the JT method [41].

**Fig. 1:**
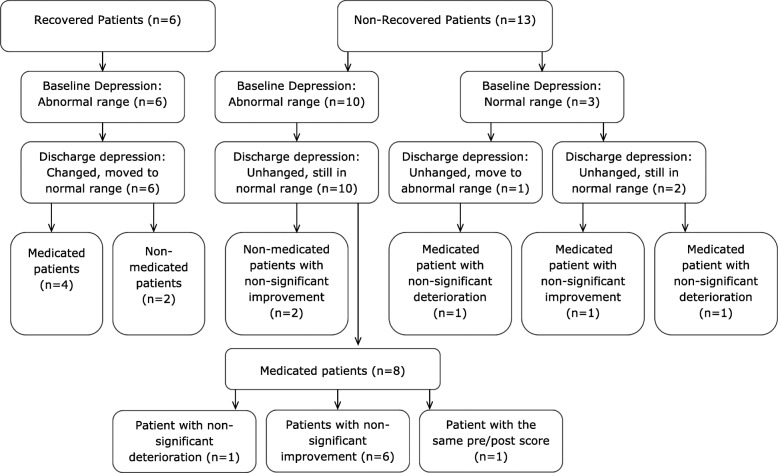
Graphical illustration of recovered and non-recovered patients

The percentage of recovered patients in the preferred intensity group increased from 32 to 38% after excluding the three minimally depressed patients, who could not technically recover due to floor effects (baseline depression scores already in the normal range). Details for the preferred intensity group are presented in Table 1 and in Fig. 1.

**Table 1:** Changes in post-intervention scores of depression in the preferred intensity exercise group

Patient Depression at Baseline (BDI-II)		Psychiatric Medication (months)	Psychological Therapies (months)	Changed recovered	Unchanged still abnormal range	Unchanged still normal range	Unchanged from normal to abnormal range
Within abnormal range							
1	Severe	120		YES			
2	Severe	36		YES			
3	Severe	84			YES ^↑^		
4	Severe	48			YES ^↑^		
5	Severe^§^	24					
6	Severe	120	192		YES ^↑^		
7	Severe	24	22		YES ^↑^		
8	Severe		24		YES ^↑^		
9	Severe		2		YES ^↑^		
10	Moderate	84		YES			
11	Moderate			YES			
12	Moderate			YES			
13	Moderate	48	12		YES ^↓^		
14	Moderate	2	2		YES ^↑^		
15	Moderate	48			YES ^↑^		
16	Mild	36		YES			
Within normal range							
17	Minimal	180				YES ^↑^	
18	Minimal	24					YES ^↓^
19	Minimal	252	24			YES ^↓^	

*BDI-II* Beck Depression Inventory-II

§: same pre-post score; ↑: non-significant improvement; ↓: non-significant deterioration

The post-intervention BDI-II data of Callaghan et al. [31] were reversely analysed (control vs. experimental group) to explore if the prescribed intensity exercise could bring about individual and clinically significant reductions in scores of depression when compared to preferred intensity exercise. Results showed only one individual in the prescribed intensity comparator (5%) demonstrated a statistically significant reduced BDI-II score at post-intervention, consistent with chance. This significant reduction was beyond the SED that was derived from the preferred intensity group. Nonetheless, this change fell within the normal population range, indicating depression recovery. Details are given in Table 2. Proportions of patients with changes in post-intervention BDI scores in the preferred or prescribed intensity groups are given in Table 3.

**Table 2:** Changes in post-intervention scores of depression in the prescribed intensity exercise group

Patient Depression at Baseline (BDI-II)		Psychiatric Medication (months)	Psychological Therapies (months)	Changed recovered	Unchanged still abnormal range	Unchanged still normal range	Unchanged from normal to abnormal range
Within abnormal range							
1	Severe	4	3		YES ^↓^		
2	Severe	102	108		YES ^↓^		
3	Severe	105			YES ^↓^		
4	Severe	3	8		YES ^↓^		
5	Severe	120	3		YES ^↓^		
6	Severe	36	36		YES ^↓^		
7	Severe				YES ^↓^		
8	Severe	6	10		YES ^↑^		
9	Severe	12			YES ^↑^		
10	Severe	72			YES ^↑^		
11	Moderate		8		YES ^↓^		
12	Moderate	36	36		YES ^↓^		
13	Moderate	36	24		YES ^↑^		
14	Moderate	32		YES			
15	Moderate				YES ^↑^		
16	Moderate	6			YES ^↑^		
Within normal range							
17	Mild^§^	105					
18	Minimal	7	12			YES ^↑^	
19	Minimal	12				YES ^↑^	

*BDI-II* Beck Depression Inventory-II

§: same pre-post score; ↑: non-significant improvement; ↓: non-significant deterioration

**Table 3:** Percentage (%) of patients with changes in post-intervention scores of depression in the preferred and prescribed intensity groups

Exercise Group	Severity of Depression at Baseline (BDI-II)	Changed Recovered (%)	Unchanged still abnormal range (%)	Unchanged still normal range (%)	Unchanged from normal to abnormal range (%)
Within abnormal range					
Preferred	Severe	22	78		
Prescribed	Severe		100		
Preferred	Moderate	50	50		
Prescribed	Moderate	17	83		
Preferred	Mild	100			
Prescribed	Mild		100		
Within normal range					
Preferred	Mild				
Prescribed	Mild			100	
Preferred	Minimal			67	33
Prescribed	Minimal			100	

*BDI-II* Beck Depression Inventory-II

Both recovered and non-recovered subgroups showed mean RPE scores indicating low intensity exercise according to exertion norms. Also, both subgroups showed mean HR scores indicating moderate intensity exercise according to the method of the age-predicted Maximum HR (MHR) (MHR = 220-age). Based on norms of the American College of Sports Medicine, RPE scores of 6–11 indicate low intensity and MHR of 64–76% indicate moderate intensity exercise (pp.165; [43]). The recovered subgroup showed a small and statistically significant correlation between HR and RPE, and statistically significant higher actual HR and lower RPE values compared to the non-recovered subgroup. Finally, there were no statistically significant differences in age between the recovered and non-recovered subgroups. Details are presented in Table 4.

**Table 4:** Characteristics of recovered (*n* = 6) and non-recovered (*n* = 13) patients in the preferred intensity group

Variable	Patients	M ± SD	
			Wilcoxon Test
HR	Recovered	109.89 ± 14.32 (66% MHR)	W(17906), *p* = 2.4e-25•
	Non-Recovered	103.11 ± 21.10 (64% MHR)	
RPE	Recovered	7.79 ± 1.87	W(46564), *p* = 1.4e-6•
	Non-Recovered	9.96 ± 2.34	
			Spearman correlation
HR RPE	Recovered	109.89 ± 14.32	.26*
		7.79 ± 1.87	
HR RPE	Non-Recovered	103.11 ± 21.10	−.05
		9.96 ± 2.34	
			Independent samples T-Test
Age	Recovered	52.67 ± 13.63	t(17) = −1.05, *p* = .30
	Non-Recovered	57.62 ± 7.15	

*MHR* Maximum Heart Rate, *M* Mean, *SD* Standard Deviation, *HR* Heart Rate, *RPE* perceived exertion

The *P*-values are Bonferroni-adjusted; *Significant at *p* < 0.001

## Discussion

This study has found that the greater antidepressant effect of the ideographic model of preferred intensity exercise over the nomothetic model of the prescribed intensity exercise seen in the RCT of Callaghan et al. [31], also corresponded to clinically significant alleviation at the individual level. It was observed that preferred intensity exercise led 32% of the group (six patients) to clinically significant reduced levels of depression post-intervention in comparison to prescribed intensity group. These changes indicated depression recovery because the statistically significant improvements also fell within the normal population range. Also, thirteen patients of the preferred group (68%) did not show a significant change in post-intervention depression, although three could not recover due to floor effects and eight showed a trend of (non-significant) improvement in depression. (Table 1 and Fig. 1). Finally, only one individual of the prescribed intensity group (5%) recorded clinically significant reduced depression post-intervention (depression recovery), as would be expected due to chance alone (Table 2).

While the original trial of Callaghan et al. [31] used group statistics, in our study we used the JT method to quantify individual/clinically significant changes in depression. Also, the JT method, in contrast to group statistics used by Callaghan et al., considers the reliability of the outcome measure, and the amount of change that thus be the result of measurement error. Hence, we clarified that the preferred vs. prescribed intensity exercise led 32% of the patients to depression recovery and no patient to deterioration in depression post-intervention.

Doose et al. [36] compared an eight week self-selected intensity exercise program to wait-for-intervention via the JT analysis [35] in depressed patients. Similar to our findings, Doose et al. [36] found that 36% of the self-selected intensity group showed recovery coefficients on the self-report depression outcome (BDI-II) post-intervention. The percentage of recovered patients increased to 63% when Doose et al. used a clinician-rated depression outcome (HAMD-17). These findings were based on intention-to-treat. Also, Doose et al. [36] recorded no patient with deterioration in depression.

Both our findings and the findings from Doose et al. [36] suggest that preferred intensity exercise brings about depression recovery and no depression inducing effects. In contrast, conventional therapies such as psychotherapy may induce deterioration in depression [44]. However, Doose et al. did not employ an active comparator, specifically the prescribed intensity exercise, nor recruited exclusively registered and female patients to provide gender specific evidence for daily practice. In response to this, we employed the active comparator of prescribed intensity and a sample exclusively consisted of female participants, as women are affected by depression and prescribed antidepressants twice as often as men [1, 45]. Also, our participants were registered patients and not media respondents. Media respondents may have a non-clinical depression despite high scores in depression checklists, and they may disclose strong outcome expectations and determination for lifestyle change. Community volunteers for exercise and depression trials are typically motivated to exercise [39]. Conversely, registered depressed patients show a more challenging profile. They have suffered persistent symptoms including psychosocial impairment that led to a health service presentation, and they often report disappointment or failure as the service use brings to the surface the disease complexity and the increased needs of the patients for systematic care [46–48].

Various studies with group-based statistical analyses have reported the affective benefits of preferred exercise in adult and adolescent general populations [21–27] and in adult and adolescent depressed patients [9, 28–30]. While prescribed intensity exercise operates via fixed intensities, preferred intensity exercise operates as a tailored intervention offering a range of intensity variations and thus, providing “space” for individual variability via personal grounds [15]. Similarly to the operation of the preferred intensity exercise, the NICE physical activity guidelines for depression ([5]; pp.8) and for primary care ([19]; pp.10) suggest individual variability components including the personal grounds of needs and preferences have to be taken into account. In line, the CANMAT guidelines recommending exercise for depression stressed the importance of self-directed over practitioner-directed therapies given that individual variability components referred to patient preferences may facilitate better treatment effects [20].

In our recovered subgroup, HR and RPE produced a small and positive significant correlation (*r* = .26), partially replicating their well-established association [16]. No such association was recorded in the non-recovered patients, possibly reflecting disturbed exertion. Indeed, depressed people show disturbed perceptual processing of information relating to exertion during exercise, as they are susceptible to adverse cognitive, behavioural and affective responses such as excessive fear or worry and exaggerated or inaccurate sensing of the intensity [16–18]. The significant (positive) correlation between HR and RPE seen only in recovered patients suggested HR and perceptual processing of information relating to exertion were, to a certain extent, counterbalanced only in recovered patients. This is supported by the fact that recovered patients exercised at a higher HR but with lower levels of exertion than their non-recovered peers. It is thus likely that the improved exertion in recovered patients is linked to a positive experience of exercise, and in turn, to depression recovery. Identifying predictors of exertion at preferred intensities may help facilitate a positive experience of exercise participation. Researchers may consider that psychological rather than physiological factors appear to be playing a salient role in exertion at moderate intensities [16, 49, 50], which our preferred intensity group selected to exercise.

### Limitations

Statistical power, intention-to-treat analysis or assessor blinding were not satisfied in our study. Such methodological flaws involve risk of bias concerns and might be linked to overestimation of treatment effects [51–53]. However, our trial, despite the pragmatic design, satisfied major internal validity criteria including low attrition (< 15%), concealed allocation or baseline balance between groups. For example, the overall internal validity scoring of our trial is 6 on the 10-item Physiotherapy Evidence Based Database scale (PEDro) [54], which evaluates low risk of bias for physical therapy interventions (e.g., exercise) using a cutoff score of ≥6. Hence, our study appears to be a low risk of bias trial according to the PEDro scale, an increasingly used scale by exercise reviews for mental [9, 55, 56] or physical health [57–59].

We did not clarify if more medicated or non-medicated patients recovered from depression. Due to the high number of medicated patients (79%) in a small sample, we could not determine if the preferred intensity exercise differentially benefitted non-medicated over medicated individuals. Two of the four non-medicated patients but only four of the fifteen medicated patients revealed recovery coefficients from depression. Our small study could not properly address this potential association. Thus, small sample size is the third limitation of our study. However, small studies represent a key limitation of the field. For example, in a recent exercise review supporting the use of aerobic exercise for depressed patients in mental health services [9] the average sample size across the reviewed trials was 41 patients and comparable to our study (*N* = 38). Finally, our original study [31] was not powered to detect between-group differences in the preferred intensity group, as these were not of primary interest in that study. Consequently, the results of the sub-group analyses in the preferred intensity group presented here, must be treated with caution.

### Strengths

Our short-term preferred intensity intervention compared to exercise on referral programs for depression (10–14 weeks; [5]) suggests the potentially key role in the early phase of depression treatment, especially since the widely used antidepressant pharmacotherapy typically requires four weeks before providing any benefit [60–62]. A pragmatic RCT for depressed adolescents recruited via health services has also found a promising antidepressant role of a six-week preferred intensity exercise with enduring effects at six-month follow-up and with cost-effective gains [29, 30, 63].

The pragmatic design of our study ensures increased external validity by replicating routine practice conditions including “real-life” settings and minimum exclusion criteria [33]. Also, this design suggests that when therapist or participant blinding is not possible, such as in exercise or psychotherapy trials, it cannot be considered a severe flaw because routine practice is also not blinded [34]. Actually, trials with blinded interventions cannot be considered fully pragmatic [32]. In addition, collateral interventions designed to support compliance, such as the psychosocial support integrated in our RCT [31], are not necessarily a bias factor in the context of pragmatic trials. Integrating a collateral intervention of this sort in a broader “complex” intervention represents what is typically seen in routine practice within a contemporary stepped collaborative care model for depression treatment [33]. Accordingly, our findings from a pragmatic trial are representative of routine practice and thus, of increased external validity.

Therefore, our findings could be considered generalizable, especially since our sample exercised in the community with safety and low attrition and represented a group which is treated mainly in primary care and affected by depression and prescribed antidepressants twice as often as men [1, 38, 45]. Moreover, our sample age (45–65) is associated with increasingly high rates in common mental health disorders including depression [4].

The final strength involves consideration that NICE [5] recommended exercise for mild or moderate depression and almost 60% of our mild or moderate depressed patients showed depression recovery (Table 1).

## Conclusion

Our secondary analysis of the pragmatic RCT of Callaghan et al. [31] via individual clinical significance analysis indicated that the ideographic model of preferred intensity exercise compared favorably to the nomothetic model of prescribed intensity exercise, as it led 32% of the patients to depression recovery four weeks post-intervention. The remaining thirteen patients of the preferred group (68%) showed no significant change in post-intervention depression; however, eight of them showed non-significant improvement in depression and three could not technically recover due to floor effects. In contrast, only one patient in the prescribed group showed a significant change in post-intervention depression (improvement), consistent with chance.

Despite our promising finding, more pragmatic RCTs are needed to employ longer-term interventions, larger female samples, follow-up evaluations and both self- and clinician-rated outcomes. In this vein, firmer conclusions can be drawn on whether preferred vs. prescribed intensity exercise can lead more depressed women to clinical significant changes in depression.

## Data Availability

The datasets used and/or analysed during the current study are available from the corresponding author on reasonable request.
